# Group precipitation and age hardening of nanostructured Fe-based alloys with ultra-high strengths

**DOI:** 10.1038/srep21364

**Published:** 2016-02-19

**Authors:** Z. B. Jiao, J. H. Luan, M. K. Miller, C. Y. Yu, C. T. Liu

**Affiliations:** 1 Center for Advanced Structural Materials, Department of Mechanical and Biomedical Engineering, College of Science and Engineering, City University of Hong Kong, Hong Kong, China; 2 Oak Ridge National Laboratory, Oak Ridge, TN 37831, USA

## Abstract

The precipitation of nanoparticles plays a key role in determining the properties of many structural materials, and the understanding of their formation and stabilization mechanisms has been a long standing interest in the material field. However, the critical issues involving the group precipitation of various nanoparticles and their cooperative hardening mechanism remain elusive in the newly discovered Fe-based alloys with nanostructures. Here we quantitatively elucidate the nucleation mechanism, evolution kinetics and hardening effects of the group-precipitated nanoparticles in the Fe-Cu-Ni-Al-based alloys by atom probe tomography together with both first-principles and thermodynamic calculations. Our results provide the compelling evidence for two interesting but complex group precipitation pathways of nanoparticles, i.e., the Cu-rich and NiAl-based precipitations. The co-existence of the two precipitation pathways plays a key role in age hardening kinetics and ultimately enhances the hardening response, as compared to the single particle type of strengthening, therefore providing an effective new approach for strengthening materials for structural applications.

Advanced structural materials, such as ultra-high strength steels, are essential to the modern world, and their continuous innovation is critical in enabling human to move towards a sustainable future^1^. With the now unprecedented environmental challenges facing mankind, it is critical to develop advanced ultra-high strength steels to dramatically reduce the amount of common low-strength steels in use at the present time. Among the various strengthening mechanisms, nanoparticles hardening has been proven to be a most effective method to enhance the strength of Fe-based alloys^2^,^3^,^4^,^5^,^6^,^7^,^8^,^9^,^10^,^11^. In particular, the group precipitation of multiple types of nanoparticles is more attractive, as compared with precipitation of a dispersion of a single type of nanoparticles, since the group precipitation approach may lead to a superior combination of different properties resulting from the synergistic combination of multiple types of nanoparticles with different compositions, microstructures, and micromechanical properties^7^,^12^. However, the critical issues involving the group precipitation of various nanoparticles and their cooperative evolution and hardening mechanisms remain to be elusive in the newly developed Fe-based ultra-high strength alloys at the present time. Furthermore, the mechanistic understanding of these issues is of fundamental importance to achieve optimal properties of these Fe-based alloys.

Recently, uniform precipitation of metallic nanoparticles, such as Cu-rich and NiAl-based nanoparticles, in ferritic steels has attracted an increasing attention, which allows the development of low-carbon steels with a good combination of high strength, good ductility, good weldability and relatively low cost^13^,^14^,^15^. It has been documented that Cu-rich nanoparticles initially have a metastable body-centered cubic (bcc) structure with diameters less than 5 nm^5^,^6^,^7^,^8^,^16^,^17^,^18^,^19^,^20^,^21^, whereas NiAl-based nanoparticles possess an ordered cubic B2 crystal structure based on the simple cubic structure with an Al atom located at the body center of a Ni cube^22^,^23^,^24^,^25^,^26^,^27^, both structures satisfying the lattice coherency requirement in the bcc α-Fe matrix. More recently, through the optimization of alloy compositions, the group precipitation of nanoscale Cu-rich and NiAl-based particles has been achieved in multi-component Fe-based alloys, which offers a promising way to effectively strengthen the Fe-based alloys to as high as 2000 MPa without a significant reduction in ductility^28^,^29^,^30^,^31^,^32^. These studies also show that small variations in the Ni, Al and Cu contents can lead to sensitive differences in the precipitation characteristics, including the composition, configuration, size, and number density of the Cu-rich and NiAl-based nanoparticles. For example, in high-Cu and low-Ni/Al alloys, Cu-rich nanoparticles nucleate first from the supersaturated solid solution, and Ni and Al tend to segregate at the interface between the Cu nanoparticles and α-Fe matrix^5^,^6^,^7^,^8^,^16^,^17^,^18^,^19^,^20^,^21^. In comparison, in low-Cu and high-Ni/Al alloys, NiAl-based nanoparticles, enriched in Ni and Al together with considerable amounts of Cu, first come out of the supersaturated solid solution^32^. To date, however, the key factors governing the nucleation mechanism and precipitation pathways of the Cu-rich and NiAl-based nanoparticles in the Fe-based alloys are not well understood, and the scientific rule for the design of high-strength alloys using the Cu-rich and NiAl-based particle strengthening has not been established yet. Thus, it is of both fundamental and practical importance to elucidate the correlation between the alloying elements, nanoscale group precipitation and hardening effects in the Fe-based alloys.

The purpose of this study is to gain insight into the synergistic alloying effects on the nanoscale group precipitation and strengthening mechanisms of the Cu-rich and NiAl-based nanoparticles on the atomic- and nano-scales, and further to provide guidelines for developing advanced Fe-based high-strength alloys with nanoparticle hardening. The precipitation characteristics, including the size, number density, morphology, spatial distribution, composition, and solute partitioning behavior of the Cu-rich and NiAl-based nanoparticles in the Fe-based alloys containing alloying additions of Cu, Ni, Al and Mn elements were thoroughly investigated by atom probe tomography (APT), which is a unique powerful tool for the characterization of composition and nanostructural features of embedded nanoparticles with a near-atomic spatial resolution and a high chemical sensitivity. In addition, thermodynamic and first-principles calculations were used to shed the light on the phase stability of the nanoparticles in these alloys. Particular attentions were paid to the understanding of the basic mechanisms governing the temporal evolution of the nanoscale group precipitates from the initial formation stages and into the growth and coarsening processes.

## Results

### Mechanical properties

**Figure 1:**
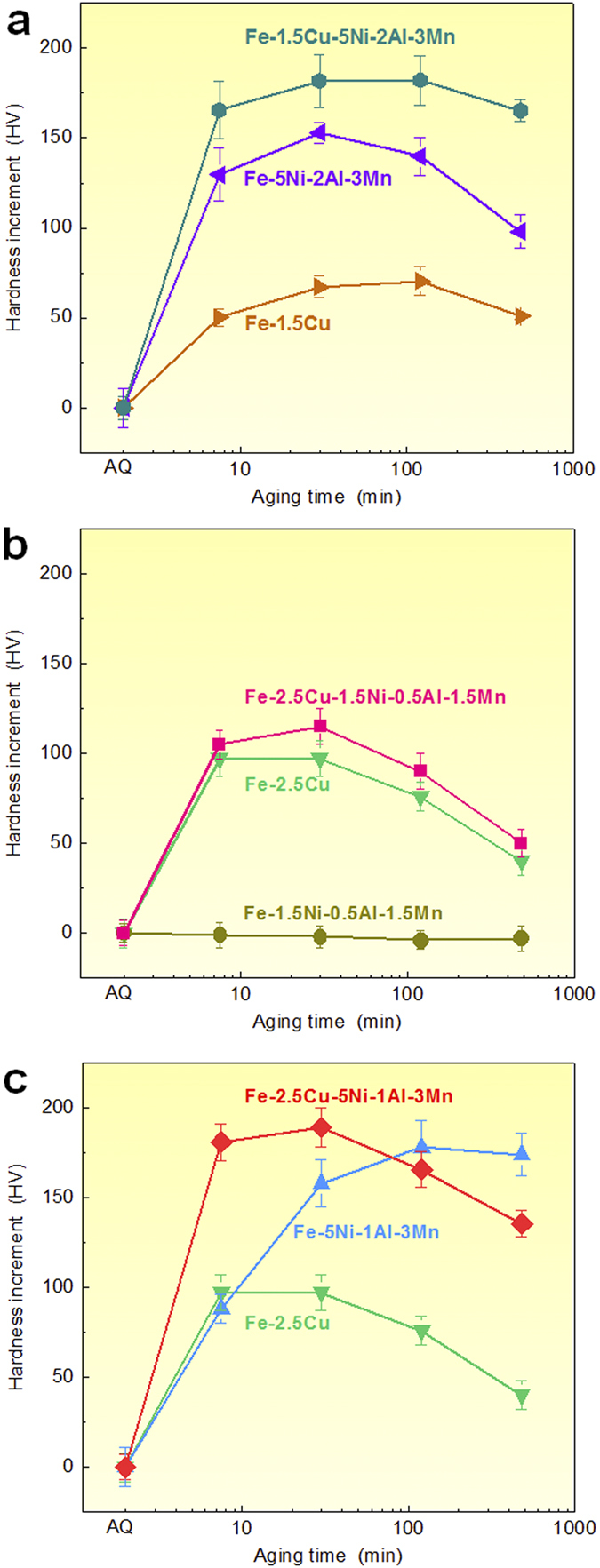
Age hardening response of the Fe-based alloys hardened by nanoparticles. (**a**) Fe-1.5Cu, Fe-5Ni-2Al-3Mn, and Fe-1.5Cu-5Ni-2Al-3Mn alloys; (**b**) Fe-2.5Cu, Fe-1.5Ni-0.5Al-1.5Mn, and Fe-2.5Cu-1.5Ni-0.5Al-1.5Mn alloys; (**c**) Fe-2.5Cu, Fe-5Ni-1Al-3Mn, and Fe-2.5Cu-5Ni-1Al-3Mn alloys.

Hardness measurements were conducted to evaluate the age response of the three types of alloys: Fe-Cu-, Fe-Ni-Al-, and Fe-Cu-Ni-Al-based alloys, the compositions of which are listed in Table 1. The Vickers hardness of these alloys are shown in the Supplementary Fig. 1s as a function of aging time at 550 °C, and the corresponding hardness increment, ∆H, relative to the base hardness in the as-quenched condition are illustrated in Fig. 1.

As shown in Fig. 1a, the Fe-1.5Cu-5Ni-2Al-3Mn alloy shows a dramatic increase of ~165 HV in hardness after aging for 7.5 min. and a further increase of ~20 HV after aging for 30 min., followed by a slight decrease due to an over-aging effect. The hardness of the Fe-5Ni-2Al-3Mn alloy also increases rapidly by ~130 HV after aging for 7.5 min., while that of the Fe-1.5Cu alloy only increases slightly by ~50 HV after aging for 7.5 min., indicating that the age hardening response of the Fe-1.5Cu alloy is much lower than that of the Fe-5Ni-2Al-3Mn and Fe-1.5Cu-5Ni-2Al-3Mn alloys.

The Fe-2.5Cu and Fe-2.5Cu-1.5Ni-0.5Al-1.5Mn alloys exhibit a similar increase in hardness within the first 7.5 min. aging period, but the hardness increment of the Fe-2.5Cu-1.5Ni-0.5Al-1.5Mn multi-component alloy is slightly higher than that of the Fe-2.5Cu binary alloy during aging from 7.5 to 480 min., as illustrated in Fig. 1b. The Fe-1.5Ni-0.5Al-1.5Mn alloy shows no appreciable changes in hardness within the 480 min. aging period, indicating that precipitation hardening does not occur in this low-Ni and low-Al alloy.

For the Fe-5Ni-1Al-3Mn alloy (Fig. 1c), the hardness increment during the first 7.5 min. shows a similar trend as observed for the Fe-2.5Cu alloy, indicating that the two alloys has a similar age hardening kinetic at the early stage of age hardening. With a combination of 2.5% Cu with 5%Ni, 1% Al and 3% Mn, the multi-component Fe-based alloy shows an even faster increase in the rate of age hardening, achieving a hardness increment of ~180 HV in the 7.5 min. condition.

The above hardness measurements demonstrate that the alloying additions of Cu, Ni and Al play an important role in determining not only the hardening response but also the age hardening kinetics. In the subsequent sections, we will focus our efforts on the mechanistic understanding of the synergistic alloying effects on the nanoscale group precipitation and age hardening in these Fe-Cu-Ni-Al-Mn alloys.

### APT characterization of the precipitation microstructures

**Figure 2:**
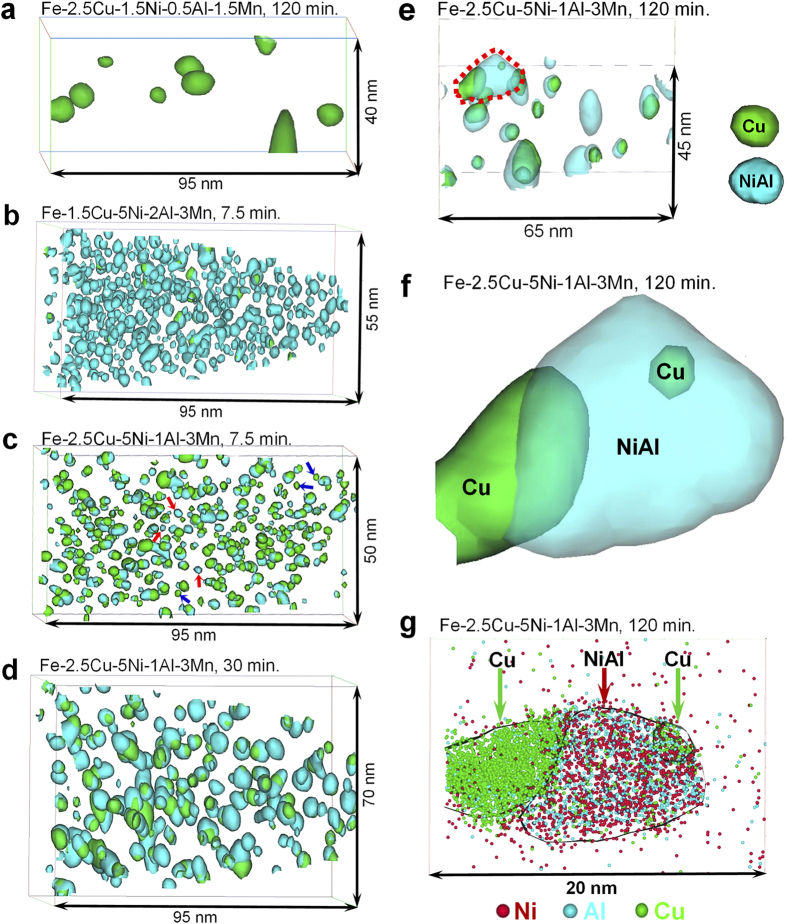
APT characterization of nanoparticles. (**a–e**) Cu-rich and NiAl-based nanoparticles in different aging conditions: **a**, Fe-2.5Cu-1.5Ni-0.5Al-1.5Mn alloy, 120 min.; (**b**) Fe-1.5Cu-5Ni-2Al-3Mn alloy, 7.5 min.; (**c**) Fe-2.5Cu-5Ni-1Al-3Mn alloy, 7.5 min.; (**d**) Fe-2.5Cu-5Ni-1Al-3Mn alloy, 30 min.; (**e**) Fe-2.5Cu-5Ni-1Al-3Mn alloy, 120 min. (**f**) Enlarged view of the red dashed section in (**e**,**g**) 1-nm-thick atom maps showing the Cu, Ni and Al atoms through the center of the Cu/NiAl co-precipitates in (**f**). For interpretation of the references to color in this figure, the reader is referred to the web version of this article.

To attain precise information about the nanoscale precipitation process, APT investigations were performed on these Fe-Cu-Ni-Al-based alloys in different aging conditions. The 10 at.% Cu concentration and 20 at.% (Ni + Al) concentration isosurfaces are used to visualize and identify the Cu-rich and NiAl-based particles, respectively. In the Fe-2.5Cu-1.5Ni-0.5Al-1.5Mn alloy (Fig. 2a), no NiAl-based nanoparticles can be observed, and only Cu-rich nanoparticle are detected in the 2 h aged condition. The average radius and number density of the Cu-rich nanoparticles are 1.40 ± 0.65 nm and 5.9 × 10^23^ m^−3^, respectively. The Cu-rich nanoparticles consist mainly of Cu (~84.23 ± 0.87 at.%) but also contain certain amounts of Ni (~3.79 ± 0.23 at.%) and Al (~3.87 ± 0.24 at.%).

In contrast, in the Fe-1.5Cu-5Ni-2Al-3Mn alloy (Fig. 2b), a large number density of NiAl-based nanoparticles and a limited number density of Cu-rich nanoparticles are detected in the 7.5 min. condition. The number density of NiAl-based nanoparticles (3.7 × 10^24^ m^−3^) is approximately an order of magnitude higher than that of Cu-rich nanoparticles (4.3 × 10^23^ m^−3^), indicating that the NiAl nanoparticles first come out of the solid solution in the aging sequence. Note that the NiAl-based nanoparticles are enriched not only in Ni (30.50 ± 0.48 at.%) and Al (25.50 ± 0.46 at.%) but also in Cu (18.00 ± 0.32 at.%), Fe (15.62 ± 0.38 at.%) and Mn (10.37 ± 0.32 at.%).

In comparison, in the Fe-2.5Cu-5Ni-1Al-3Mn alloy, a high number density of both Cu-rich and NiAl-based nanoparticles are detected readily in the aged specimens. This alloy was selected as an example to study the morphological and compositional evolution of the two types of nanoparticles in different aging conditions. The nanostructures of the Cu-rich and NiAl-based nanoparticles in the Fe-2.5Cu-5Ni-1Al-3Mn alloy after aging for 7.5, 30, and 120 min. are shown in Fig. 2c–e. It is interesting to find that the majority of the Cu-rich and NiAl-based nanoparticles are co-precipitated with each other, and only a small number of Cu-rich and NiAl-based nanoparticles are isolated particles with no other type of nanoparticles associated with them. The isolated Cu-rich and NiAl-based nanoparticles with small sizes are labeled by blue and red arrows, respectively, in Fig. 2c. In the 120 min. condition, a ~8 nm NiAl-based particle is neighboring to two Cu-rich particles, the left with a size of ~6 nm and the right of ~2 nm, as illustrated in the circled area in Fig. 2e. The enlarged view of the whole structure of the three particles is displayed in Fig. 2f, and the 1-nm-thick atom map for Ni, Al and Cu atoms through the center of the three particles are shown in Fig. 2g, in which the relative positions and extents of the Al (cyan), Ni (red) and Cu (green) atoms are indicated. The distinction between the coarse Cu-rich and NiAl-based particles is clear from the isoconcentration surface, but the fine Cu-rich particle (the right-upper one labeled by the green arrow) is largely overlapped with the NiAl-based particle. The fine Cu-rich particle contains a relatively lower amount of Cu but a higher amount of Ni and Al, as compared with the coarse one. Considering the relatively small size and low Cu content in it, the fine Cu-rich particle is likely to be a newly formed nanoparticle.

**Figure 3:**
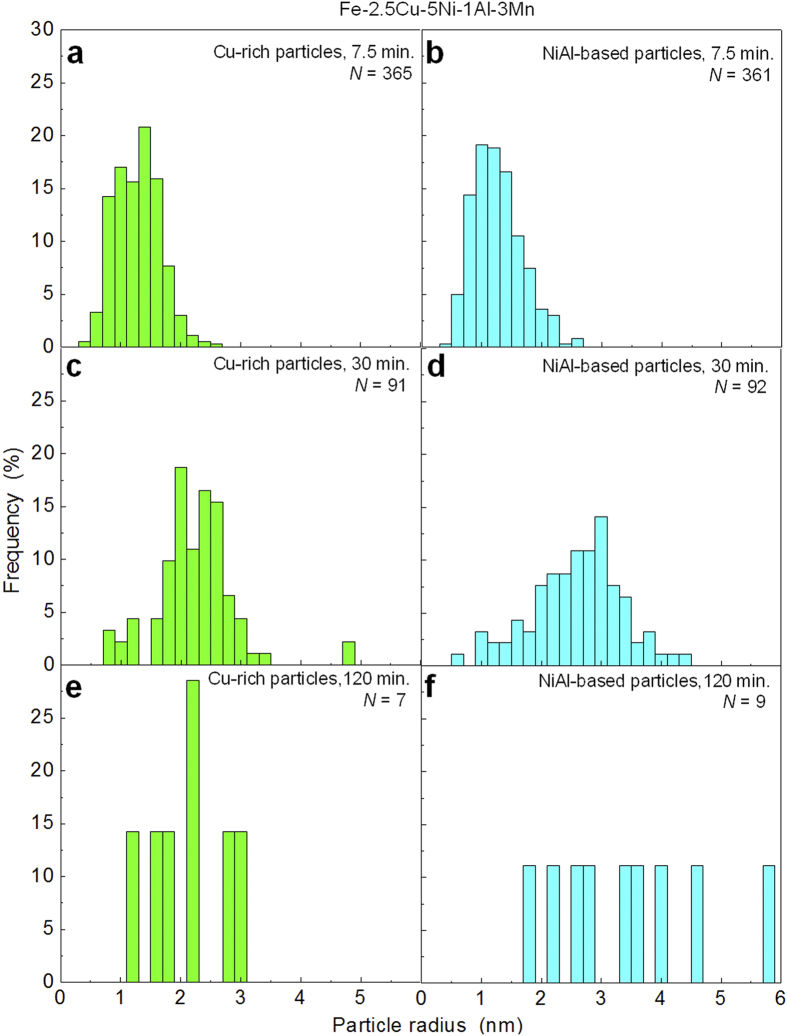
Particle size distributions of the Cu-rich and NiAl-based nanoparticles in different aging conditions. (**a**) Cu-rich particles, 7.5 min.; (**b**) NiAl-based particles, 7.5 min.; (**c**) Cu-rich particles, 30 min.; (**d**) NiAl-based particles, 30 min.; (**e**) Cu-rich particles, 120 min.; (**f**) NiAl-based particles, 120 min.

The size distributions of the Cu-rich and NiAl-based nanoparticles in different aging conditions are shown in Fig. 3. As the aging time increases, the size distribution of both the Cu-rich and NiAl-based nanoparticles becomes wider and shifts to slightly larger particle sizes. The statistical information, including the average particle radius and number density, as a function of aging time is summarized in Table 2. In the 7.5 min. condition, the Cu-rich and NiAl-based nanoparticles exhibit a similar particle size and number density. The average radius and number density of the Cu-rich nanoparticles are ~1.28 ± 0.38 nm and ~1.4 × 10^24^ m^−3^, respectively, and that of the NiAl-based nanoparticles are ~1.27 ± 0.41 nm and ~1.4 × 10^24^ m^−3^, respectively. As the aging time increases to 30 min., a slight increase in average radius and a slight decrease in number density are observed in both types of nanoparticles. The average radii of the Cu-rich and NiAl-based nanoparticles increase to ~2.24 ± 0.66 and ~2.59 ± 0.75 nm, respectively, while their number densities decrease to ~4.2 × 10^23^ and ~4.7 × 10^23^ m^−3^, respectively. With a further increasing aging time to 120 min., the Cu-rich and NiAl-based nanoparticles coarsen significantly, and the low number densities of the coarse particles in the long-term aged condition result in a small number of particles in similar volumes of analysis. For a qualitative understanding of the precipitation microstructure in the 120 min. condition, the average radius and number density of the Cu-rich nanoparticles are estimated to be ~2.23 ± 0.64 nm and ~1.6 × 10^23^ m^−3^, respectively, and that of the NiAl-based nanoparticles are estimated to be ~3.40 ± 1.23 nm and ~2.3 × 10^23^ m^−3^, respectively.

### Compositional evolution of the Cu-rich and NiAl-based nanoparticles

**Figure 4:**
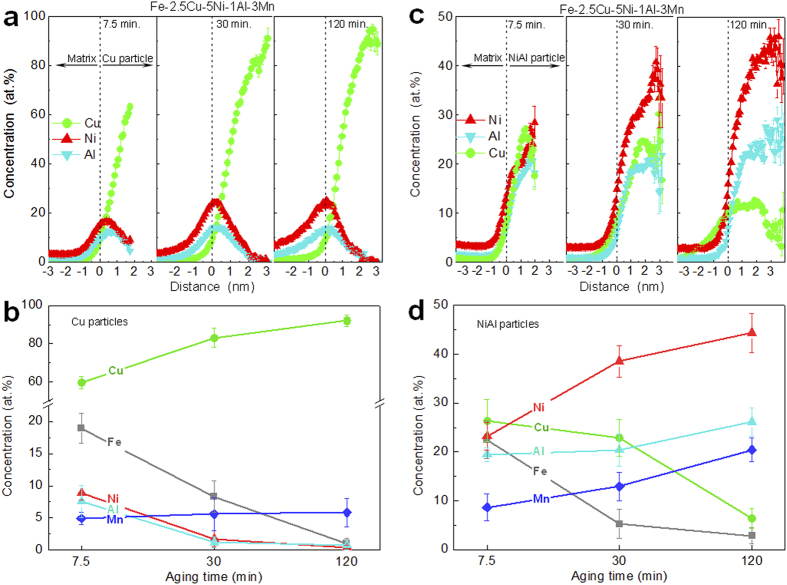
Proximity histograms and compositions of the Cu-rich and NiAl-based nanoparticles. (**a**) Proximity histograms of the Cu-rich particles; (**b**) Compositions of the Cu-rich particles; (**c**) Proximity histograms of the NiAl-based particles; (**d**) Compositions of the NiAl-based particles. The proximity histograms are the average of the individual proximity histograms for all the particles in the data.

The quantification of the solute partitioning between the nanoparticles and the matrix was determined from proximity histograms^33^, in which the compositional information with respect to the distance from the particle/matrix interface is presented. The proximity histograms of the Cu-rich particles in Fe-2.5Cu-5Ni-1Al-3Mn alloy after aging for 7.5, 30 and 120 min. are illustrated in Fig. 4a, which are the average of the individual proximity histograms for all the Cu-rich particles in the data. The quantitative composition analysis of all the Cu-rich particles from the proximity histograms is summarized in Fig. 4b. In all three aging conditions, the enrichment of Cu change monotonically towards the center of the Cu-rich particles, whereas Ni and Al exhibit a local enrichment at the Cu-rich particle/matrix interface. After 7.5 min., the Cu-rich particles are enriched in Cu (59.69 ± 3.02 at.%) but also contain significant amounts of Fe (18.94 ± 2.33 at.%), Ni (8.90 ± 1.13 at.%), Al (7.54 ± 2.49 at.%), and Mn (4.93 ± 0.92 at.%). As the aging time increases, the Cu concentration in the Cu-rich particles increases to 83.11 ± 5.10 at.% at 30 min. and achieves a value of 92.00 ± 2.97 at.% at 120 min., whereas the concentrations of Ni and Al decrease continuously in the Cu-rich particles, reaching values of 0.39 ± 0.33 at.% and 0.78 ± 0.73 at.%, respectively, at 120 min. Meanwhile, the degree of the interfacial segregation of Ni and Al increases significantly with aging time, from 15.64 ± 0.11 at.% Ni and 9.92 ± 0.09 at.% Al at 7.5 min. to 24.51 ± 0.46 at.% Ni and 13.86 ± 0.37 at.% Al at 120 min. In the 7.5 min. condition, the concentration peak of Ni (16.62 ± 0.20 at.%) is located at a distance of 0.35 nm, whereas that of Al (12.53 ± 0.24 at.%) is observed more close to the particle center at a distance of 0.75 nm. In the 120 min. condition, a pronounced enrichment of Ni and Al is observed at almost the same location, close to the Cu-rich particle/matrix interface.

The proximity histograms of the NiAl-based particles in Fe-2.5Cu-5Ni-1Al-3Mn alloy after aging for 7.5, 30 and 120 min. are illustrated in Fig. 4c, which are the average of the individual proximity histograms for all the NiAl-based particles in the data. The quantitative composition analysis of all the NiAl-based particles from the proximity histograms is summarized in Fig. 4d. The concentration profiles of Ni and Al across the NiAl-based particle/matrix interface are increased monotonically, with the maximum occurring near the center of the NiAl-based particles, while that of Cu exhibits a non-monotonic behavior. After aging for 7.5 min., the NiAl-based particles contain 23.21 ± 2.82 at.% Ni, 19.43 ± 1.45 at.% Al, 8.60 ± 2.73 at.% Mn, 26.35 ± 4.33 at.% Cu and 22.47 ± 3.83 at.% Fe. The concentrations of Ni and Al increase to 38.56 ± 3.21 and 20.36 ± 3.35 at.%, respectively, after aging for 30 min., and further increase to 44.36 ± 3.99 and 26.12 ± 2.90 at.%, respectively, after aging for 120 min., whereas that of Cu decreases significantly with aging time, from 26.35 ± 4.33 at.% at 7.5 min. to 6.32 ± 2.01 at.% at 120 min. (by a factor of ~4). It is interesting to pointed out that the Mn concentration increases from 8.60 ± 2.73 at.% at 7.5 min. to 20.39 ± 2.44 at.% at 120 min., leading to the formation of the Ni(Al,Mn) nanoparticles. As the role of Mn in promoting the formation of the Ni(Al,Mn) nanoparticles has been discussed in detail in ref. 27, it is not discussed repeatedly in this paper. Furthermore, at the shorter aging times (7.5 and 30 min.), no significant segregation of Cu to the NiAl-based particle/matrix interface was observed. However, after the longer aging time (120 min.), a broad concentration peak is located near the NiAl-based particle/matrix interface, where the Cu concentration (10.15 ± 0.23 at.%) is higher than that in the center of the NiAl-based particles (6.32 ± 2.01 at.%) and that in the matrix (0.28 ± 0.02 at.%).

### First-principles calculations of sublattice occupancies in NiAl-based nanoparticles

**Figure 5:**
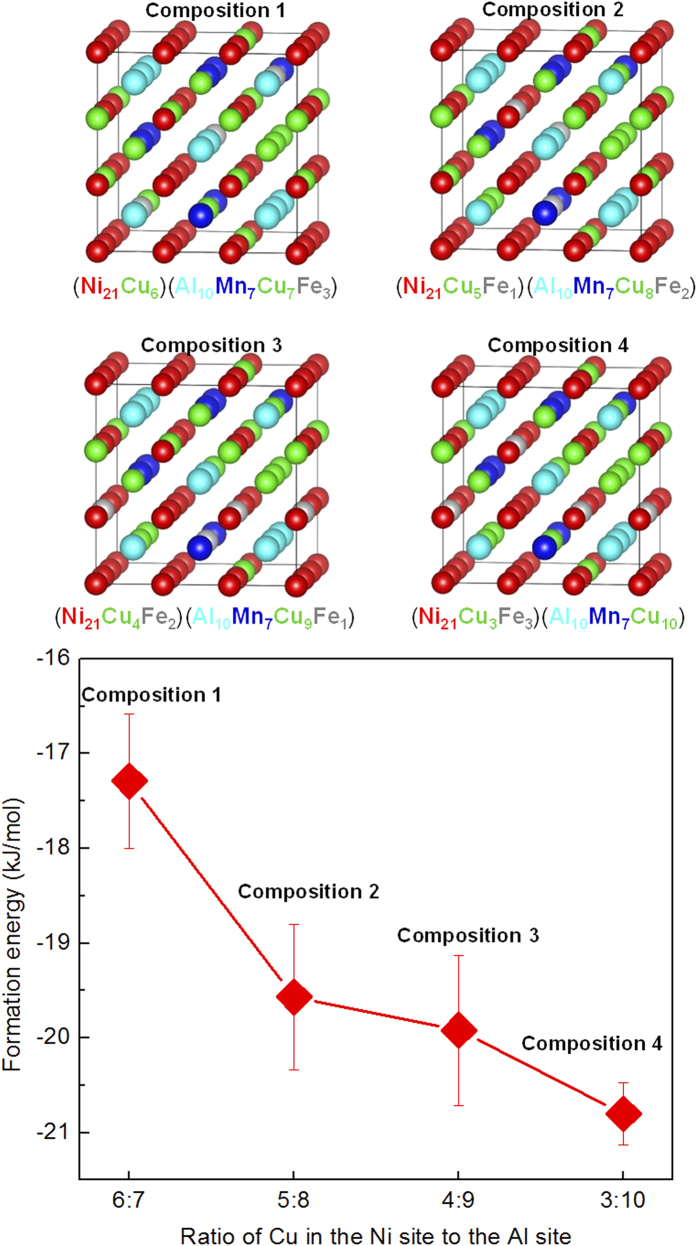
Atomic structures and calculated formation energies of the NiAl-based models with different sublattice occupancies of Cu and Fe. Model 1: (Ni_21_Cu_6_)(Al_10_Mn_7_Cu_7_Fe_3_), model 2: (Ni_21_Cu_5_Fe_1_)(Al_10_Mn_7_Cu_8_Fe_2_), model 3: (Ni_21_Cu_4_Fe_2_)(Al_10_Mn_7_Cu_9_Fe_1_), and model 4: (Ni_21_Cu_3_Fe_3_)(Al_10_Mn_7_Cu_10_). For interpretation of the references to color in this figure, the reader is referred to the web version of this article.

Previous both experimental and computational studies had indicated that Mn preferentially occupy the Al sublattice^27^,^34^, whereas Cu and Fe can go to both the Ni and Al sublattices in B2-ordered NiAl-type intermetallic alloys, depending on the specific alloy composition^35^,^36^. To explore the delicate sublattice occupancy of Cu and Fe in the multi-component NiAl-based nanoparticles, the first-principles calculations were performed in the 54-atom supercells comprising 21 Ni, 10 Al, 7 Mn, 13 Cu and 3 Fe atoms, where the atomic ratios between all these elements are based on the composition of the NiAl-based particles obtained by aging at 550 °C for 30 min. analyzed by APT (38.56Ni-20.36Al-12.97Mn-22.92Cu-5.25Fe, at.%). The 54-atom supercells were constructed based on the NiAl-type BCC lattice, in which 17 Al atoms were substituted by Mn, Cu and Fe atoms randomly, and 6 Ni atoms were substituted by Cu and Fe atoms randomly according to our specific configuration. Four B2-ordered NiAl-type compositions with different substitutional sites were constructed and illustrated in Fig. 5: (1) (Ni_21_Cu_6_)(Al_10_Mn_7_Cu_7_Fe_3_), (2) (Ni_21_Cu_5_Fe_1_)(Al_10_Mn_7_Cu_8_Fe_2_), (3) (Ni_21_Cu_4_Fe_2_)(Al_10_Mn_7_Cu_9_Fe_1_), and (4) (Ni_21_Cu_3_Fe_3_)(Al_10_Mn_7_Cu_10_), in which the ratio of Cu in the Ni site to the Al site are 6:7, 5:8, 4:9 and 3:10, respectively. The formation energies of the multi-component nanoparticles with different Cu and Fe sublattice occupancies were calculated according to the following equation:


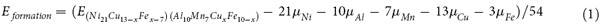


where 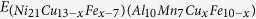 is the total energies of the 54-atom supercells with different Cu and Fe sublattice occupancies, *x* = 7, 8, 9 and 10, representing the number of Cu atoms in the Al site of the 54-atom supercells, and *μ*_*Ni*_, *μ*_*Al*_, *μ*_*Mn*_, *μ*_*Cu*_ and *μ*_*Fe*_ are the chemical potentials of the Ni, Al, Mn, Cu and Fe elements in their ground-state elemental form, respectively. Spin-polarized calculations were performed for those with magnetic ordering. In view of the importance of configurational averting of supercells^37^, 4 configurations were calculated for each composition in order to obtain a reliable estimation. Based on the calculated total energies and chemical potentials in the 54-atom supercells, the formation energies of the four compositions with different Cu and Fe substitution sites were obtained and are shown in Fig. 5. For (Ni_21_Cu_6_)(Al_10_Mn_7_Cu_7_Fe_3_), with 54% of the Cu occupying the Al site (composition 1), the formation energy is determined to be −17.29 ± 0.71 kJ/mol. As more Cu occupies the Al site, the formation energy decreases gradually. When the majority of the Cu occupies the Al site (composition 4), the formation energy of (Ni_21_Cu_3_Fe_3_)(Al_10_Mn_7_Cu_10_) decreases to −20.80 ± 0.33 kJ/mol. The above calculations indicate that the majority of the Cu occupying the Al site and all of the Fe occupying the Ni site is energetically favored, implying that Cu is essentially substitutional for the Al site and Fe for the Ni site in the NiAl-based nanoparticles.

## Discussion

The results presented above indicate that, upon aging, the nanoscale Cu-rich and NiAl-based nanoparticles undergo a rather complex interaction and evolution, involving the changes in both composition and morphology. For an understanding and eventually control over the complex group precipitation behavior, it is of fundamental importance to elucidate the nucleation mechanism and precipitation pathways of the two types of nanoparticles.

The nucleation behavior of the Cu-rich and NiAl-based nanoparticles in the Fe-based alloys during aging isothermally at 550 °C was analyzed first. The APT results in Fig. 2 show that the nucleation sequence of the Cu-rich and NiAl-based particles in the Fe-Cu-Ni-Al-based alloys is highly sensitive to the Cu, Ni and Al contents. In the high-Cu and low-Ni/Al alloys, such as the Fe-2.5Cu-1.5Ni-0.5Al-1.5Mn (wt.%) alloy, the Cu-rich particles nucleate first from the supersaturated solid solution (Fig. 2a), whereas in the low-Cu and high-Ni/Al alloys, such as the Fe-1.5Cu-5Ni-2Al-3Mn (wt.%) alloy, the NiAl-based particles nucleate first from the supersaturated solid solution (Fig. 2b). In comparison, in the Fe-2.5Cu-5Ni-1Al-3Mn alloy with both the high Cu and high Ni/Al contents, a small number of both isolated Cu-rich and NiAl-based nanoparticles with small sizes can be detected by APT at the early stage of precipitation (as labeled by blue and red arrows, respectively, in Fig. 2c), indicating that both Cu-rich and NiAl-based particles can nucleate independently from the supersaturated solid solution in this alloy system. The extremely high number densities of particles (10^23^−10^24^ m^−3^) would indicate a homogeneous rather than heterogeneous type of precipitation simply based on the large number of nucleation sites required, which would be much larger than presented by a normal or even high dislocation density or any other heterogeneous microstructural features. The concurrent homogeneous nucleation of Cu-rich and NiAl-based particles may be attributed to relatively both the high Cu and high Ni/Al contents, which provides the thermodynamic possibility for the independent nucleation of Cu-rich and NiAl-based particles. To understand the thermodynamic origins of the concurrent formation of the Cu-rich and NiAl-based particles, thermodynamic calculations were performed to calculate the solubility of Cu and NiAl in the α-Fe, using the Thermo-Calc software with the Fe-based database (TCFE7). The equilibrium solubility of Cu in the α-Fe is estimated to be ~0.15 wt.% at 550 °C. The studied alloy contains 2.5 wt.% Cu, indicating a high chemical driving force for the Cu-rich precipitation. However, the Thermo-Calc software with the TCFE7 database cannot predict the formation of the B2-ordered NiAl phase due to the lack of a database resource. Alternatively, we experimentally evaluated the minimum Ni and Al contents required for the NiAl precipitation in the α-Fe, and our studies showed that a minimum of 2.5 wt.% Ni and 1 wt.% Al is required^26^. In the Fe-2.5Cu-5Ni-1Al-3Mn alloy, the concentrations of Ni far exceeds the minimum requirement, thus also having a high driving force for the precipitation of the NiAl phase. In addition, the hardness profiles in Fig. 1c also show that both the Fe-2.5Cu and Fe-5Ni-1Al-3Mn alloys exhibit significant age hardening responses upon aging at 550 °C, suggesting that the additions of 2.5% Cu and a combination of 5% Ni, 1% Al and 3% Mn are sufficiently high enough for the independent nucleation of Cu-rich and NiAl-based nanoparticles from the α-Fe matrix, respectively.

Next the precipitation pathways of the Cu-rich and NiAl-based nanoparticles was elucidated. It is interesting to point out that although both the Cu-rich and NiAl-based nanoparticles can form independently from the supersaturated solid solution, generically, it is more favorable for them to heterogeneously precipitate at beside the other type of pre-existing particles, both resulting in the formation of the Cu/NiAl co-precipitates. This may be an important reason for the APT observation that the majority of the Cu-rich and NiAl-based particles are co-precipitated with each other. In the following, the mechanisms of the two precipitation pathways, i.e., the Cu-rich and NiAl-based precipitation, as well as their synergistic effects on the nanoscale group precipitation behavior in Fe-based alloys will be elucidated.

First, the mechanism of the NiAl-based precipitation and its influence on the Cu-rich precipitation are discussed. As mentioned above, the Fe-2.5Cu-5Ni-1Al-3Mn alloy contains sufficient Ni and Al concentrations beyond the miscibility gap for disordered bcc-Fe and B2-ordered NiAl, which makes the independent nucleation of NiAl in the α-Fe matrix possible. Interestingly, at the early stage of precipitation, the NiAl nanoparticles are enriched not only in Ni and Al but also in Cu (~26.35 ± 4.33 at.% at 7.5 min). It is known that the lattice constant of B2-NiAl (0.2886 nm) is larger than that of the α-Fe (0.2864 nm, ~0.8%), so the formation of NiAl nanoparticles would create an elastic misfit strain at the NiAl-based particle/matrix interface^24^. In this study, our first-principles calculations reveal that Cu is energetically favorable to occupy the Al sublattice and Fe preferentially occupies the Ni sublattice, thereby forming a metastable (Ni,Fe)(Al,Mn,Cu) phase. It is known that Cu (1.28 Å) has a smaller atomic size than Al (1.43 Å), and Fe (1.22 Å) has a smaller atomic size than Ni (1.24 Å)^38^. Thus, it is interesting to point out that the sublattice occupancy of Cu on the Al site and Fe on the Ni site would decrease the lattice misfit strain between the (Ni,Fe)(Al,Mn,Cu) phase and the α-Fe matrix, thereby reducing the misfit strain energy of the (Ni,Fe)(Al,Mn,Cu) particles. In addition, the lattice parameters of the 54-atom supercells with different sublattice occupancies were also determined by the DFT simulations. The compositions from 1 to 4 (Fig. 5) are determined to be 2.8875 ± 0.0016, 2.8837 ± 0.0026, 2.8785 ± 0.0016 and 2.8764 ± 0.0022 Å, respectively. That is, the lattice parameter of the supercells decreases gradually with more Cu occupying the Al sublattice and more Fe occupying the Ni sublattice, supporting the above statement about the lattice mismatch minimization via the sublattice occupancy. As the precipitation reaction proceeds, the Cu concentration in the NiAl nanoparticles shows a significant decrease with aging time, from ~26.35 ± 4.33 at.% at 7.5 min. to ~6.32 ± 2.01 at.% at 120 min. Also, the position of the Cu concentration peak moves from near the center of the NiAl-based nanoparticles to near the NiAl-based particle/matrix interface, suggesting a substantial rejection of Cu atoms outwards from the NiAl-based nanoparticles. The rejection of Cu solutes from the NiAl-based particles can be explained by the positive heat of mixing of the Cu-Ni pair (+4 kJ/mol) as the driving force for phase separation^39^. As the equilibrium solubility of Cu in the α-Fe phase is negligibly small (~0.15 wt.% at 550 °C), even smaller than that in the NiAl phase, it is unlikely that the Cu atoms, being rejected outwards from the NiAl-based particles, have any chance to be released back into the supersaturated solid-solution matrix because no increase of Cu concentration in the matrix is detected by APT. Instead, these Cu atoms, being rejected from the (Ni,Fe)(Al,Mn,Cu) particles, are favorable to diffuse into the Cu-rich particles, either by entering the existing Cu-rich particles adjacent to the NiAl-based particles or forming new Cu-rich particles near the NiAl-based particle/matrix interface. An example of the heterogeneous precipitation of a new Cu-rich particle from the NiAl-based particles is shown in Fig. 2g (the right one labeled by the green arrow). The fine Cu-rich particle, partially encased by the NiAl-based particle, contains relatively larger amounts of Ni and Al, as compared with the coarse Cu-rich particle (the left one labeled by the green arrow in Fig. 2g) in this condition, indicating this fine Cu-rich particle is formed by the rejection of Cu solutes from the NiAl-based particles. Therefore, the partitioning of Cu atoms into the Cu-rich particles have two sources in the later stages of precipitation; one is the diffusion of Cu atoms from the supersaturated solid-solution matrix and the other is the rejection of Cu solutes from the NiAl-based particles. From the above, the synergistic alloying effects in the NiAl-based precipitation can be summarized as follows: Cu atoms, enriched in the NiAl-type nanoparticles at the early stage of precipitation, can effectively reduce the strain energy of the NiAl-type particles. As the precipitation reaction proceeds, the Cu atoms, being rejected from the NiAl-based particles, would diffuse into the Cu-rich particles, acting as an atom source for the Cu-rich precipitation in addition to the supersaturated solid-solution matrix.

Secondly, the mechanism of the Cu-rich precipitation and its effect on the NiAl-based precipitation at a later stage are discussed. The Cu concentration in the Fe-2.5Cu-5Ni-1Al-3Mn alloy is approximately 16 times higher than the solubility limit of Cu in the α-Fe, resulting in a high chemical driving force for the Cu-rich precipitation. Interestingly, Ni and Al atoms are also initially enriched in the Cu-rich nanoparticles at 7.5 min. (8.90 ± 1.13 and 7.54 ± 2.49 at.%, respectively), but the majority of these two elements are then rejected to the Cu-rich particle/matrix interface after aging for 30 min. The rejection of the Ni and Al atoms outwards from the Cu-rich nanoparticles can be explained by the heat of mixing between these elements. The mixing enthalpy for the Ni-Cu pair is positive (+4 kJ/mol), indicating a tendency to solute rejection between them, while that for the Ni-Al pair is large negative (−21 kJ/mol), indicating a tendency to solute attraction between them^39^. Therefore, the co-segregation of Ni and Al at the Cu-rich particle/matrix interface can be observed. It should be pointed out that the segregation of Ni and Al atoms at the Cu-rich particle/matrix interface is initiated from not only the rejection of the two elements from the center of the Cu-rich particles but also the diffusion of the two elements from the supersaturated solid solution matrix. The beneficial effects of the interfacial segregation of Ni and Al can be twofold. First, it has been found that the interfacial segregation of Ni and Al can effective reduce the interfacial energy of the Cu-rich particles, thereby significantly promoting the Cu-rich precipitation^5^,^6^,^7^,^8^,^16^,^17^,^18^,^19^,^20^,^21^. Second, the enrichment of Ni and Al at the Cu-rich particle/matrix interface can lead to the heterogeneous nucleation of NiAl-based particles. At the early stage of precipitation, a large amount of NiAl particles with a radius less than 1 nm are located at the Cu-rich particle/matrix interface, suggesting that the Cu-rich particle/matrix interface can be a preferred nucleation site for the formation of the NiAl-based particles. According to the classical nucleation theory^40^, the heterogeneous nucleation at the pre-existing interfaces has a much lower nucleation barrier as compared with the homogeneous nucleation in the matrix, and the local enrichment of Ni and Al at the Cu-rich particle/matrix interface can provide a high chemical driving force for the NiAl-based particle nucleation. Thus, the heterogeneous nucleation of NiAl-based particles at the Cu-rich particle/matrix interface is energetically favorable, allowing for a fast precipitation of NiAl-based particles, as compared with the homogeneous precipitation of NiAl-based particles. Therefore, the segregation of Ni and Al at the Cu-rich particle/matrix interface plays an important role not only in reducing the interfacial energy of the Cu-rich particles but also in promoting the heterogeneous nucleation of the NiAl-based particles.

**Figure 6:**
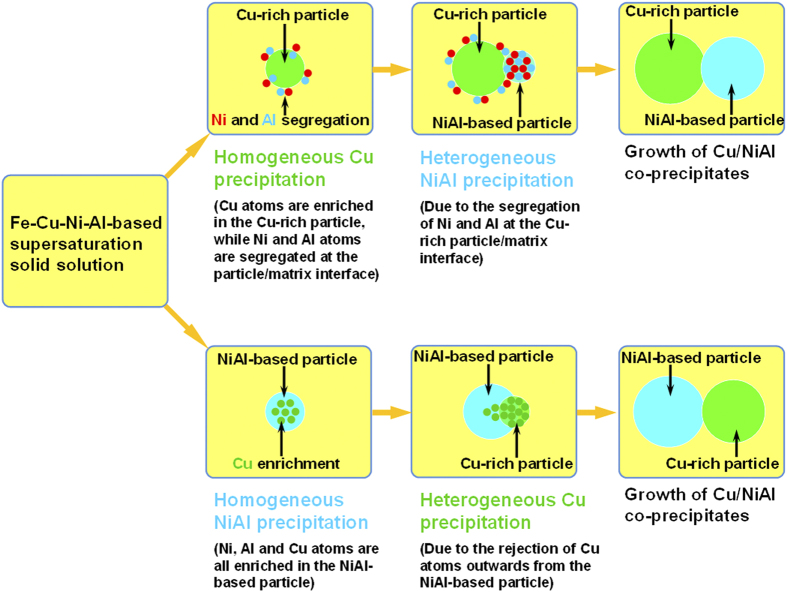
Schematics showing the two types of precipitation pathways of nanoscale Cu/NiAl co-precipitates. The upper pathway shows “supersaturated solid solution → Cu-rich particles → Cu-rich particles + NiAl-based particles”, and the lower pathway presents “supersaturated solid solution → NiAl-based particles → NiAl-based particles + Cu-rich particles”. For interpretation of the references to color in this figure, the reader is referred to the web version of this article.

Based on all the above discussion on the nucleation and evolution of the Cu-rich and NiAl-based nanoparticles, the mechanism of the Cu-rich and NiAl-based precipitation processes can be summarized, and the two types of precipitation pathways, i.e., “supersaturated solid solution → Cu-rich particles → Cu-rich particles + NiAl-based particles” “supersaturated solid solution → NiAl-based particles → NiAl-based particles + Cu-rich particles” are schematically illustrated in Fig. 6. In the first type of pathway (the upper one in Fig. 6), the Cu-rich particles, enriched in Cu together with a considerable amount of Ni and Al, nucleate first from the supersaturated solid solution. As the particles grow, both Ni and Al tend to segregate at the Cu-rich particle/matrix interface, and their enrichment leads to the heterogeneous precipitation of NiAl-based particles at the Cu-rich particle/matrix interface, thereby forming the Cu/NiAl co-precipitates. In the second type of pathway (the lower one in Fig. 6), the NiAl-based particles, enriched in Ni and Al together with a significant amount of Cu, nucleate first from the supersaturated solid solution. As the precipitation reaction proceeds, the first formation of the NiAl-based particles leads to the rejection of Cu solutes outwards from the NiAl-based particles, which results in the heterogeneous precipitation of Cu-rich particles on the outer surface of the NiAl-based particles, also forming the Cu/NiAl co-precipitates.

Finally, the implications of the group precipitation of Cu-rich and NiAl-based nanoparticles on the hardening effects and alloy design of high-strength Fe-based alloys are discussed. Since the degree of nanoparticle strengthening is strongly dependent upon the type, size and number density of the particles, the processes of the Cu-rich and NiAl-bases precipitation have to be controlled in order to obtain the desired properties. One benefit of the group precipitation of Cu-rich and NiAl-based nanoparticles is the acceleration of the age hardening kinetics. As illustrated in Fig. 6, the first formation of Cu-rich particles would promote the precipitation of NiAl due to the segregation of Ni and Al at the Cu-rich particle/matrix interface, while the first formation of NiAl-based particles would also promote the precipitation of Cu-rich particles by rejecting Cu outward from the NiAl-based particles. Thus, the unusual co-existence of the two types of precipitation pathways, i.e., “supersaturated solid solution → Cu-rich particles → Cu-rich particles + NiAl-based particles” and “supersaturated solid solution → NiAl-based particles → NiAl-based particles + Cu-rich particles”, can lead to the formation of extremely high number densities of both Cu-rich and NiAl-based particles within a relatively short time. For example, the hardness of the Fe-2.5Cu-5Ni-1Al-3Mn alloy is close to its peak hardness after aging for 7.5 min., while that of the Fe-5Ni-1Al-3Mn alloy without any Cu additions can only reach half of its peak hardness in the same aging condition. The shortening of the aging time due to the group precipitation is of a great practical relevance and a distinctive cost advantage from the view of materials processing. Another benefit of the group precipitation of Cu-rich and NiAl-based nanoparticles is the ability to balance the materials properties with cost. Although the ordered NiAl-based nanoparticles with a high ordering energy^41^,^42^,^43^ have a higher strengthening efficiency than the soft Cu-rich nanoparticles, the cost of Ni is much higher than that of Cu. A proper combination of strengthening from Cu-rich and NiAl-based particles can lead to the development of ultra-high strength steels with a relatively low Ni content^28^,^29^,^30^,^31^,^32^, as compared with the maraging steels (e.g., Fe-5Co-19Ni-1Al-5Mo-1Cr, wt.%) hardened only by NiAl-based particles^44^. It should be pointed out, however, that the group precipitation of Cu-rich and NiAl-based nanoparticles is not always the most appropriate strategy to strengthen alloys. One unusual but interesting phenomenon of the group precipitation approach is the development of the insufficient thermal stability of the co-precipitates. As shown in Fig. 1c, the Fe-2.5Cu-5Ni-1Al-3Mn alloy overages faster than the Fe-5Ni-1Al-3Mn alloy hardened only by the NiAl-based nanoparticles. The possible reason for the fast coarsening of the co-precipitates may be due to the solute transfer between the Cu-rich and NiAl-based particles. The proximity histograms in Fig. 4a show that as the precipitation proceeds, both Ni and Al are rejected outwards from the Cu-rich particles and enriched at the Cu-rich particle/matrix interface. These Ni and Al atoms can directly be incorporated in the NiAl-based particles adjacent to the Cu-rich particles, thereby increasing the size of the NiAl-based particles and accelerating their coarsening rate. The thermal stability of nanostructures is practically important for the design of high-strength materials for elevated temperature and irradiation applications^2^,^3^,^4^,^45^,^46^. The aging profiles in Fig. 1c indicate that the NiAl-based particles, rather than the Cu/NiAl co-precipitates, may be a good choice for strengthening at elevated-temperatures, and some successful applications of NiAl-based nanoparticles in ferritic superalloys have been reported recently^3^,^24^,^25^. By illustrating the above key factors governing the age hardening kinetics and hardening response, the present study provides useful guidelines for the development of Fe-based high-strength alloys using the Cu-rich and NiAl-based particles hardening.

In summary, the synergistic alloying effects on the group precipitation and age hardening of the Cu-rich and NiAl-based nanoparticles were thoroughly studied by APT together with both first-principles and thermodynamic calculations. Two types of interesting but complex group precipitation processes of nanoparticles containing mainly Cu, Ni and Al atoms are observed. One is the homogeneous Cu-rich precipitation, followed by the heterogeneous NiAl-based precipitation adjacent to the Cu-rich particles due to the interfacial segregation of Ni and Al atoms, and the other is the homogeneous NiAl-based precipitation, followed by the heterogeneous Cu-rich precipitation adjacent to the NiAl-based particles due to the rejection of Cu solutes. The unusual co-existence of the two types of precipitation pathways and the synergistic alloying effects significantly accelerate the age hardening kinetics and ultimately enhance the hardening response as compared with the single particle type of strengthening, therefore providing an effective approach for strengthening materials for structural applications. This finding not only provides new insights into the understanding of the nanoscale group precipitation and strengthening mechanisms in Fe-based alloys, but also has important implications in designing advanced high-strength alloys by engineering the nanostructural features.

## Methods

The alloys were prepared by arc-melting a mixture of commercially pure metals (purity > 99.8 wt.%) in a Ti-gettered high-purity argon atmosphere. Repeated melting was performed at least five times to improve the chemical homogeneity of the alloy. The melted alloys were then drop-cast into a copper mold with dimensions of 50 × 15 × 6 mm. The resulting plates were cold rolled by multiple passes with a total reduction of ~66% and solution-treated for 30 min. at 900 °C, followed by water quenching, and then aged isothermally at 550 °C for various periods of time up to 32 h for precipitation reactions. Hardness measurements were conducted using a Vickers hardness tester with a load of 2 N for 15 s, and for each specimen, at least 8 indents were measured to obtain an average value.

The needle-shaped specimens for APT were fabricated by a combination of standard electropolishing methods followed by an annular mill in a FEI Nova 200 focused-ion beam/scanning electron microscope^20^. The APT characterizations were performed in a CAMECA Instruments LEAP 4000× HR local electrode atom probe. The specimen were analyzed in voltage mode with a specimen temperature of 50 K, a pulse repetition rate of 200 kHz, a pulse fraction of 0.2, and an ion collection rate of between 0.5% and 1% ions per field evaporation pulse. Imago Visualization and Analysis Software version 3.6.6 was used for three-dimensional reconstruction, composition analyses, and the creation of isoconcentration surfaces.

Equilibrium solubility were calculated using the software Thermo-Calc 3.0.1, together with an Fe-based database (TCFE7). The first-principles calculations were based on the density functional theory implemented in the Vienna ab initio Simulation Package (VASP)^47^,^48^. Projector augmented wave (PAW)^49^,^50^ potential and the generalized gradient approximation (GGA) of Perdew-Burke-Ernzerhof^51^ were used to describe the coulomb interaction of ion cores with the valence electrons and the electronic exchange and correlation, respectively. Three-dimensional 54-atom periodic supercells with 3 × 3 × 3 unit cells were used to determine the total energies, with a plane-wave energy cutoff of 400 eV and 3 × 3 × 3 Γ-centered Monkhorst-Pack grids^52^. Equilibrium cell volumes and all internal atomic positions of the supercells were fully relaxed until convergence with the total energy tolerance of 10^−4^ eV.

## Additional Information

**How to cite this article**: Jiao, Z. B. *et al.* Group precipitation and age hardening of nanostructured Fe-based alloys with ultra-high strengths. *Sci. Rep.* **6**, 21364; doi: 10.1038/srep21364 (2016).

## Supplementary Material

Supplementary Information

## Figures and Tables

**Table 1:** Alloy compositions of the Fe-Cu-, Fe-Ni-Al- and Fe-Cu-Ni-Al-based alloys (wt.%).

Alloy	Cu	Ni	Al	Mn	Fe
Fe-1.5Cu	1.5	—	—	—	Balance
Fe-5Ni-2Al-3Mn	—	5.0	2.0	3.0	Balance
Fe-1.5Cu-5Ni-2Al-3Mn	1.5	5.0	2.0	3.0	Balance
Fe-2.5Cu	2.5	—	—	—	Balance
Fe-1.5Ni-0.5Al-1.5Mn	—	1.5	0.5	1.5	Balance
Fe-2.5Cu-1.5Ni-0.5Al-1.5Mn	2.5	1.5	0.5	1.5	Balance
Fe-5Ni-1Al-3Mn	—	5.0	1.0	3.0	Balance
Fe-2.5Cu-5Ni-1Al-3Mn	2.5	5.0	1.0	3.0	Balance

**Table 2:** Particle radius and number density of the Cu-rich and NiAl-based particles in the Fe-2.5Cu-5Ni-1Al-3Mn alloy in different aging conditions.

Condition	Particle	Particle radius (nm)	Number density (1/m^3^)
7.5 min. at 550 °C	Cu-rich	1.28 ± 0.38	1.4 × 10^24^
7.5 min. at 550 °C	NiAl-based	1.27 ± 0.41	1.4 × 10^24^
30 min. at 550 °C	Cu-rich	2.24 ± 0.66	4.2 × 10^23^
30 min. at 550 °C	NiAl-based	2.59 ± 0.75	4.7 × 10^23^

## References

[b1] Lu K. The future of metals. Science 328, 319–320 (2010). 20395503 10.1126/science.1185866

[b2] Trotter G., Rayner G., Baker I. & Munroe P. R. Accelerated precipitation in the AFA stainless steel Fe–20Cr–30Ni–2 Nb–5Al via cold working. Intermetallics 53, 120–128 (2014).

[b3] Huang S. *et al.* Deformation mechanisms in a precipitation-strengthened ferritic superalloy revealed by *in situ* neutron diffraction studies at elevated temperatures. Acta Mater. 83, 137–148 (2015).

[b4] Hirata A. *et al.* Atomic structure of nanoclusters in oxide-dispersion-strengthened steels. Nature Mater. 10, 922–926 (2011). 22019943 10.1038/nmat3150

[b5] Fine M. E. & Isheim D. Origin of copper precipitation strengthening in steel revisited. Scr. Mater. 53, 115–118 (2005).

[b6] Fine M. E. *et al.* A New Paradigm for Designing High-Fracture-Energy Steels. Metall. Mater. Trans. A 41, 3318–3325 (2010).

[b7] Mulholland M. D. & Seidman D. N. Nanoscale co-precipitation and mechanical properties of a high-strength low-carbon steel. Acta Mater. 59, 1881–1897 (2011).

[b8] Kolli R. P. & Seidman D. N. The temporal evolution of the decomposition of a concentrated multicomponent Fe–Cu-based steel. Acta Mater. 56, 2073–2088 (2008).

[b9] Hayashi T., Sarosi P. M., Schneibel J. H. & Mills M. J. Creep response and deformation processes in nanocluster-strengthened ferritic steels. Acta Mater. 56, 1407–1416 (2008).

[b10] Millán J. *et al.* Designing Heusler nanoprecipitates by elastic misfit stabilization in Fe–Mn maraging steels. Acta Mater. 76, 94–105 (2014).

[b11] Kim S. H., Kim H. & Kim N. J. Brittle intermetallic compound makes ultrastrong low-density steel with large ductility. Nature 518, 77–79 (2015). 25652998 10.1038/nature14144

[b12] Sun Z. *et al.* New design aspects of creep-resistant NiAl-strengthened ferritic alloys. Scr. Mater. 68, 384–388 (2013).

[b13] Czyryca E. J. Advances in high strength steel technology for naval hull construction. Key Eng. Mater. 84, 491–520 (1993).

[b14] Taillard R. & Pineau A. Room temperature tensile properties of Fe-19wt.% Cr alloys precipitation hardened by the intermetallic compound NiAl. Mater. Sci. Eng. 56, 219–231 (1982).

[b15] Calderon H. A., Fine M. E. & Weertman J. R. Coarsening and morphology of β′ particles in Fe-Ni-Al-Mo ferritic alloys. Metall. Trans. A 19, 1135–1146 (1988).

[b16] Othen P. J., Jenkins M. L. & Smith G. D. W. High-resolution electron microscopy studies of the structure of Cu precipitates in α-Fe. Philo. Mag. A 70, 1–24 (1994).

[b17] Monzen R., Iguchi M. & Jenkins M. L. Structural changes of 9 R copper precipitates in an aged Fe-Cu alloy. Philo. Mag. Lett. 80, 137–148 (2000).

[b18] Isheim D., Gagliano M. S., Fine M. E. & Seidman D. N. Interfacial segregation at Cu-rich precipitates in a high-strength low-carbon steel studied on a sub-nanometer scale. Acta Mater. 54, 841–849 (2006).

[b19] Miller M. K., Wirth B. D. & Odette G. R. Precipitation in neutron-irradiated Fe–Cu and Fe–Cu–Mn model alloys: a comparison of APT and SANS data. Mater. Sci. Eng. A 353, 133–139 (2003).

[b20] Miller M. K. & Russell K. F. Embrittlement of RPV steels: An atom probe tomography perspective. J. Nucl. Mater. 371, 145–160 (2007).

[b21] Jiao Z. B. *et al.* Synergistic effects of Cu and Ni on nanoscale precipitation and mechanical properties of high-strength steels. Acta Mater. 61, 5996–6005 (2013).

[b22] Miller M. K. & Hetherington M. G. Atom probe analysis of β‘ precipitation in a model iron-based Fe-Ni-Al-Mo superalloy. J. de Phys. 50, C8–425 (1989).

[b23] Yamamoto Y. *et al.* Creep-resistant, Al_2_O_3_-forming austenitic stainless steels. Science 316, 433–436 (2007). 17446398 10.1126/science.1137711

[b24] Teng Z. K. *et al.* Characterization of nanoscale NiAl-type precipitates in a ferritic steel by electron microscopy and atom probe tomography. Scr. Mater. 63, 61–64 (2010).

[b25] Vo N. Q., Liebscher C. H., Rawlings M. J., Asta M. & Dunand D. C. Creep properties and microstructure of a precipitation-strengthened ferritic Fe–Al–Ni–Cr alloy. Acta Mater. 71, 89–99 (2014).

[b26] Jiao Z. B., Luan J. H., Zhang Z. W., Miller M. K. & Liu C. T. High-strength steels hardened mainly by nanoscale NiAl precipitates. Scr. Mater. 87, 45–48 (2014).

[b27] Jiao Z. B., Luan J. H., Miller M. K., Yu C. Y. & Liu C. T. Effects of Mn partitioning on nanoscale precipitation and mechanical properties of ferritic steels strengthened by NiAl nanoparticles. Acta Mater. 84, 283–291 (2015).

[b28] Kapoor M. *et al.* Aging characteristics and mechanical properties of 1600 MPa body-centered cubic Cu and B2-NiAl precipitation-strengthened ferritic steel. Acta Mater. 73, 56–74 (2014).

[b29] Zhang Z. *et al.* A nanoscale co-precipitation approach for property enhancement of Fe-base alloys. Sci. Rep. 3, 1237 (2013). 23429646 10.1038/srep01327 PMC3579184

[b30] Wen Y. R. *et al.* Microstructure characterization of Cu-rich nanoprecipitates in a Fe-2.5 Cu-1.5 Mn-4.0 Ni-1.0 Al multicomponent ferritic alloy. Acta Mater. 61, 2133–2147 (2013).

[b31] Wang X., Sha G., Shen Q. & Liu W. Age-hardening effect and formation of nanoscale composite precipitates in a NiAlMnCu-containing steel. Mater. Sci. Eng. A 627, 340–347 (2015).

[b32] Jiao Z. B., Luan J. H., Miller M. K. & Liu C. T. Precipitation mechanism and mechanical properties of an ultra-high strength steel hardened by nanoscale NiAl and Cu particles. Acta Mater. 97, 58–67 (2015).

[b33] Hellman O. C., Vandenbroucke J. A., Rüsing J., Isheim D. & Seidman D. N. Analysis of three-dimensional atom-probe data by the proximity histogram. Microsc. Microanal. 6, 437–444 (2000). 11003678 10.1007/S100050010051

[b34] Kolli R. P., Mao Z., Seidman D. N. & Keane D. T. Identification of a Ni_0.5_(Al_0.5−x_Mn_x_) B2 phase at the heterophase interfaces of Cu-rich precipitates in an α-Fe matrix. Appl. Phys. Lett. 91, 241903 (2007).

[b35] Anderson I. M., Duncan A. J. & Bentley J. Site-distributions of Fe alloying additions to B2-ordered NiAl. Intermetallics 7, 1017–1024 (1999).

[b36] Bozzolo G., Mosca H. O., Wilson A. W., Noebe R. D. & Garces J. E. Atomistic modeling of quaternary alloys: Ti and Cu in NiAl. Metall. Mater. Trans. B 33, 265–284 (2002).

[b37] Lumley S. C. *et al.* The thermodynamics of hydride precipitation: The importance of entropy, enthalpy and disorder. Acta Mater. 79, 351–362 (2014).

[b38] Gale W. F. & Totemeier T. C. (Eds.). Smithells metals reference book (Butterworth-Heinemann, London, 2003).

[b39] Takeuchi A. & Inoue A. Classification of bulk metallic glasses by atomic size difference, heat of mixing and period of constituent elements and its application to characterization of the main alloying element. Mater. Trans. 46, 2817–2829 (2005).

[b40] Kelton K. & Greer A. L. Nucleation in condensed matter: applications in materials and biology Vol. 15 (Elsevier, Amsterdam, 2010).

[b41] Kumar K. S., Mannan S. K. & Viswanadham R. K. Fracture toughness of NiAl and NiAl-based composites. Acta Metall. Mater. 40, 1201–1222 (1992).

[b42] Bei H., Shim S., Pharr G. M. & George E. P. Effects of pre-strain on the compressive stress–strain response of Mo-alloy single-crystal micropillars. Acta Mater. 56, 4762–4770 (2008).

[b43] Thompson R. J., Zhao J. C. & Hemker K. J. Effect of ternary elements on a martensitic transformation in β-NiAl. Intermetallics 18, 796–802 (2010).

[b44] Ohishi K., Uehara T. & Kishigami I. *Maraging steel.* U.S. Patent Application 14/348, 308 (2012).

[b45] Stoica G. M., Stoica A. D., Miller M. K. & Ma D. Temperature-dependent elastic anisotropy and mesoscale deformation in a nanostructured ferritic alloy. Nat. Commun. 5, 5178 (2014). 25300893 10.1038/ncomms6178

[b46] Chookajorn T., Murdoch H. A. & Schuh C. A. Design of stable nanocrystalline alloys. Science 337, 951–954 (2012). 22923577 10.1126/science.1224737

[b47] Kresse G. & Furthmüller J. Efficiency of ab-initio total energy calculations for metals and semiconductors using a plane-wave basis set. Comput. Mater. Sci. 6, 15–50 (1996). 10.1103/physrevb.54.11169 9984901

[b48] Kresse G. & Furthmüller J. Efficient iterative schemes for ab initio total- energy calculations using a plane-wave basis set. Phys. Rev. B 54, 11169–11186 (1996). 10.1103/physrevb.54.11169 9984901

[b49] Blöchl P. E. Projector augmented-wave method. Phys. Rev. B 50, 17953–17979 (1994). 10.1103/physrevb.50.17953 9976227

[b50] Kresse G. & Joubert D. From ultrasoft pseudopotentials to the projector augmented-wave method. Phys. Rev. B 59, 1758–1775 (1999).

[b51] Perdew J. P., Burke K. & Ernzerhof M. Generalized Gradient Approximation Made Simple. Phys. Rev. Lett. 77, 3865–3868 (1996). 10062328 10.1103/PhysRevLett.77.3865

[b52] Monkhorst H. J. & Pack J. D. Special points for Brillouin-zone integrations. Phys. Rev. B 13, 5188–5192 (1976).

