# Supplementary material for: Group precipitation and age hardening of nanostructured Fe-based alloys with ultra-high strengths
Source: Sci Rep. 2016 Feb 19;6:21364. doi: 10.1038/srep21364 (PMC4759528; doi:10.1038/srep21364)
Supplement: Supplementary Information [file srep21364-s1.doc]

**Supplementary Information for**

**Group precipitation and** **age hardening of nanostructured** **Fe-based alloys with ultra-high strengths**

Z. B. Jiao^1^, J. H. Luan^1^, M. K. Miller^2^, C. Y. Yu^1^, C. T. Liu^1^,[[1]](#footnote-2)

^1^ Center for Advanced Structural Materials, Department of Mechanical and Biomedical Engineering, College of Science and Engineering, City University of Hong Kong, Hong Kong, China

^2^Oak Ridge National Laboratory, Oak Ridge, TN 37831, USA

**Supplementary Figure**


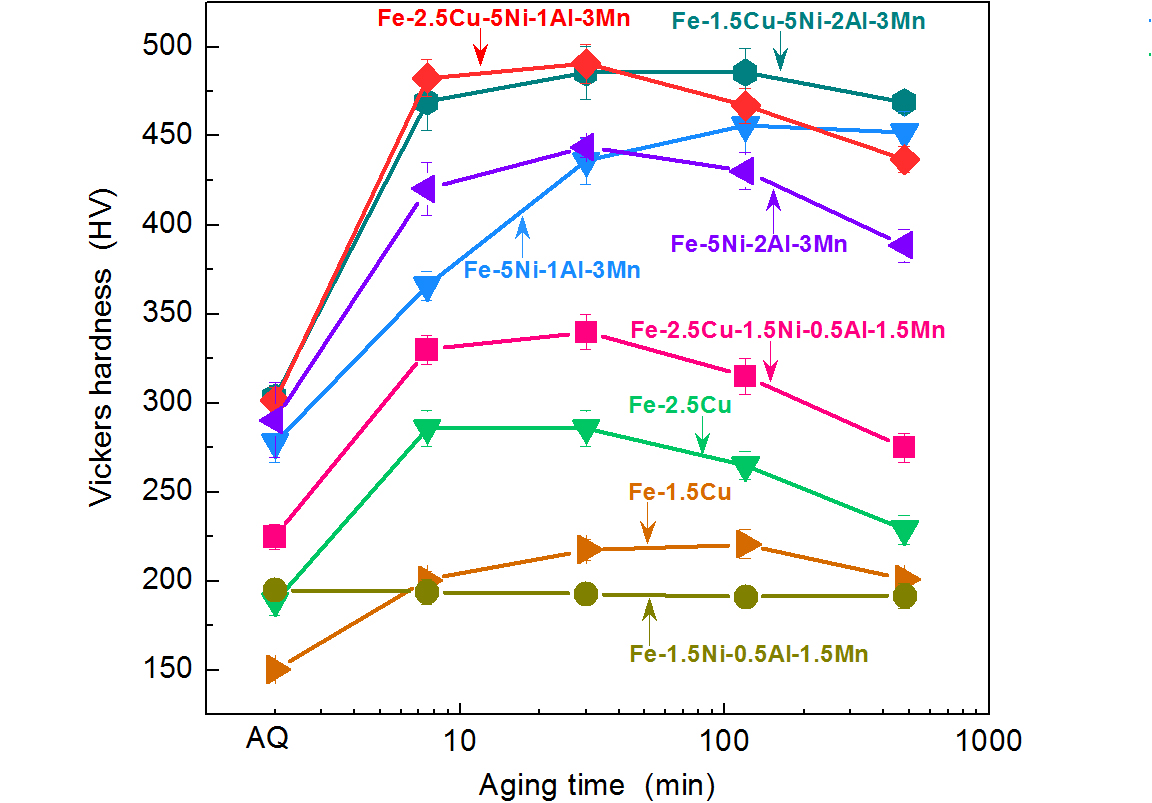


**Fig. 1s.** **Hardness as a function of aging time for the Fe-Cu-, Fe-Ni-Al- and Fe-Cu-Ni-Al-based alloys.**

1. Corresponding author, E-mail: [chainliu@cityu.edu.hk](mailto:chainliu@cityu.edu.hk), Tel: 852-34427213, Fax: 852-34420172 [↑](#footnote-ref-2)
